# Enhanced Therapeutic Epidermal Growth Factor Receptor (EGFR) Antibody Delivery via Pulsed Ultrasound with Targeting Microbubbles for Glioma Treatment

**DOI:** 10.1007/s40846-015-0032-9

**Published:** 2015-04-10

**Authors:** Ai-Ho Liao, Hsin-Yi Chou, Yi-Lei Hsieh, Sheng-Chieh Hsu, Kuo-Chen Wei, Hao-Li Liu

**Affiliations:** Graduate Institute of Biomedical Engineering, National Taiwan University of Science and Technology, Taipei, 106 Taiwan; Department of Electrical Engineering, Chang Gung University, Taoyuan, 333 Taiwan; Department of Biomedical Sciences, College of Medicine, Chang Gung University, Taoyuan, 333 Taiwan; Department of Neurosurgery, Chang Gung University and Memorial Hospital, Linkou, Taoyuan, 333 Taiwan; Medical Imaging Research Center, Institute for Radiological Research and Health Aging Research Center, Chang Gung University, Taoyuan, 333 Taiwan

**Keywords:** Microbubbles, Epidermal growth factor receptor, Pulsed ultrasound

## Abstract

Pulsed-mode ultrasound (pUS) in combination with intravenously (IV) administered microbubbles (MBs) can enhance local drug delivery by temporarily enhancing capillary permeability. This study evaluates the use of epidermal growth factor receptor (EGFR)-targeting MBs after pUS treatment to enhance the effects of therapeutic-EGFR antibody delivery to glioma tumor cells in mice. Three animal groups were compared: (1) IV-injected non-targeting MBs, (2) IV-injected targeting MBs, and (3) IV-injected targeting MBs combined with pUS treatment. All animals were analyzed using high-frequency small-animal US imaging. The mean halftime of circulating targeting MBs was significantly increased from 3.13 min of targeting bubble alone to 5.86 min by targeting MBs combined with pUS treatment, compared to 2.34 min for non-targeting MBs. Compared to targeting bubble administration alone, pUS exposure prior to injection of targeting MBs was also significantly better at suppressing tumor growth when monitored for up to 35 days (*p* < 0.05). The final relative tumor volumes were 2664, 700, and 188 mm^3^ for non-targeting MBs, targeting MBs, and targeting MBs combined with pUS treatment, respectively. pUS treatment prolonged the mean circulatory halftime of targeting MBs and enhanced the anti-tumor effect of EGFR antibodies in a human glioma model in mice. Targeting MBs combined with pUS treatment thus has potential for enhanced therapeutic antibody delivery for facilitating anti-glioma treatment.

## Introduction

Epidermal growth factor receptor (EGFR) is a single transmembrane receptor tyrosine kinase. The binding of a ligand, such as epidermal growth factor (EGF) or transforming growth factor-α (TGF-α), to the extracellular domain of EGFR induces its conformational change, dimerization, and trans-phosphorylation of specific receptor tyrosine residues. These phosphorylated-tyrosine residues generate docking sites for downstream signaling molecules, leading to the activation of phosphoinositide 3 kinase/AKT, mitogen-activated protein (MAP) kinase, and JNK-STATs pathways. These signaling activities promote cell proliferation, mobility, and anti-apoptosis [1, 2]. The mechanisms involved in the activation of EGFR include receptor overexpression [3], autocrine activation by overproduction of ligands [4], ligand-independent activation through other receptor systems and mutant receptors resulting in ligand-independent activation [5]. Preclinical and clinical studies have validated the targeting of EGFR as an anticancer therapy. Four treatment strategies that involve the targeting of EGFR and the blocking of its downstream signaling pathways have been developed: monoclonal antibodies directed against the extracellular domain of EGFR; small molecules for intracellular blocking of tyrosine-kinase activation (tyrosine-kinase inhibitors, TKIs); antisense oligonucleotides to inhibit EGFR synthesis; and antibody-based immunoconjugates such as immunotoxins or immunoliposomes for specific and efficient delivery of anticancer agents to EGFR-overexpressing tumors [6].

Malignant gliomas constitute the most common primary brain tumors in adults and rank among the most devastating and aggressive types of human cancer due to their dismal prognosis. A number of genetic alterations are responsible for the malignancy of these tumors, and mutations leading to the hyperactivation of receptor tyrosine kinases are common. EGFR is often overexpressed and amplified in gliomas, and contributes to uncontrolled proliferation and survival of glioma cells [7]. EGFR inhibition is effective in the reduction of the proliferation, motility, and invasion of cells expressing wild-type, mutant, or amplified EGFR [3].

Pulsed-mode ultrasound (pUS) exposure therapy was recently shown to enhance the anti-tumor effect of an EGFR-targeting chemotherapeutic drug in colon-cancer-bearing mice [8]. pUS shows great potential for the targeted delivery of therapeutic agents, including chemotherapeutic drugs, nanoparticles, and genes, to tumors [9]. Gas-filled echogenic microbubbles (MBs) are applied as a clinical contrast agent to enhance vascular information in US imaging. pUS-enhanced local brain drug delivery also relies on the intravenous (IV) administration of MBs and interaction with ultrasonic energy for the temporary disruption of the lumen and endothelial cell junction to enhance permeability of tissues to circulating drugs. pUS in the presence of MBs (pUS-MBs) is a noninvasive technique that shows great promise for local and reversible enhancement of the permeability of central nervous system (CNS) capillaries (so-called blood–brain barrier disruption) to enhance chemotherapeutic agent delivery in malignant glioma applications [10]. Imaging the tumor vasculature can be clinically useful for both the diagnosis and monitoring of tumor responses to antiangiogenic therapy. MBs have also been used as drug carriers and thus provide the potential to combine US imaging with pUS-mediated therapy [11, 12].

EGFR therapeutic antibodies such as cetuximab and panitumumab can inhibit the binding of ligands to EGFR and suppress EGFR signaling, and have been approved by the FDA for the treatment of head and neck cancer and metastatic colorectal cancer [13]. Cetuximab induces internalization of the antibody-receptor complex leading to down-regulation of EGFR expression. Furthermore, the Fc region of the antibody may recruit and activate immune cells or complement to induce antibody-dependent cell-mediated cytotoxicity or complement-dependent cytotoxicity [14]. Pre-clinical studies have also shown that cetuximab treatment can promote radiation-induced apoptosis and inhibit tumor angiogensis [15].

**Fig. 1:**
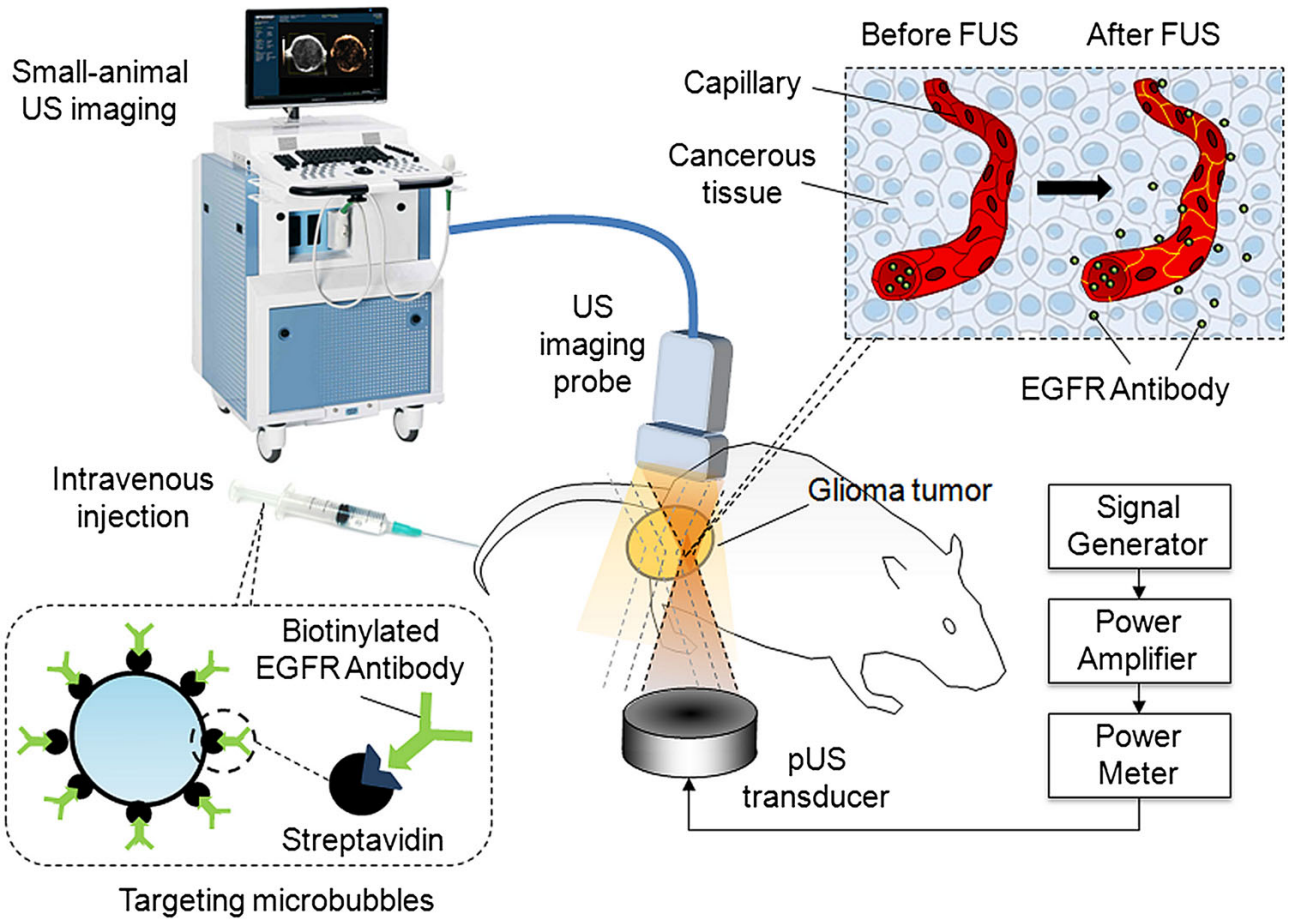
Conceptual diagram of this study. pUS beam was focused on glioma tumor. After injection of targeting MBs, US imaging was performed

The present study evaluates the potential of pUS exposure combined with therapeutic EGFR-targeting MB administration for glioma treatment (concepts shown in Fig. 1). It is hypothesized that pUS-MB exposure can enhance blood-tumor permeability and prolong the circulation of the targeting MBs and enhance the local concentration of therapeutic EGFR delivery at the local tumor site to provide therapeutic efficacy.

## Materials and Methods

### Human Primary Glioblastoma Animal Model

U87 glioma cells were cultured at 37 °C in an incubator containing 5 % CO_2_ in humidified air. Tumor cells were harvested for culture or injection by washing the monolayer with phosphate-buffered saline (PBS), followed by a brief incubation in 0.25 % trypsin and 0.02 % ethylenediaminetetraacetic acid (EDTA) to detach the cells. After removal of the trypsin and EDTA, 0.1 ml of cell suspension containing 1 × 10^6^ tumor cells in PBS was injected via a 27-gauge needle into the hypoderm of the right-lower limbs of male BALB/c nude mice (Bio-LASCO, Taipei, Taiwan). All animal experiments were performed according to an animal care protocol approved by the National Animal Center of Taiwan. All mice were maintained according to the regulations of Chang Gung University’s Institutional Animal Care and Use Committee. BALB/c nude mice were raised in a room with the thermostat set to 26 °C. Mice were 5–6 weeks old and weighed 25–30 g. Tumor size was measured every 4 days after tumor implantation until the diameter of the tumor reached about 8 mm or could be firmly fixed for monitoring.

### Preparation of MBs

Targestar™-SA (Targeson, La Jolla, CA, USA) MBs were used in this study to serve as the non-targeting MBs as well as the US contrast agent to evaluate image intensity. Targestar™-SA MBs were suspended in aqueous saline at a concentration of approximately 1 × 10^9^ particles/ml, and had a median diameter of approximately 2.5 µm. Targestar™-SA MBs conjugated with EGFR antibody (Thermo Fisher Scientific Anatomical Pathology, CA, USA) by biotin-streptavidin conjugation was used for either US imaging or therapy and served as the targeting MBs. The conjugated monoclonal antibodies can block the EGF- and TGFα-induced activation of EGFR (but have no effect on tyrosine kinase activity of receptor I) and have been confirmed to block tumor growth in vivo [16–18].

### In Vivo High-Frequency US Imaging of MBs

A previous study demonstrated the usefulness of using high-frequency US to monitor tumor perfusion and micro-environmental changes [19]. U87-tumor-bearing male BALB/c nude mice (*n* = 6 in each group) were kept anesthetized with 2 % isoflurane in oxygen at 2 l/min on a scanning stage. The hair on the skin over the tumor was clipped, and acoustic gel was applied to the tumor. A commercial small-animal US imaging system (Vevo 2100, VisualSonics, Toronto, Canada) was used. The array transducer had a central frequency of 18 MHz, with axial and lateral resolutions of 75 and 165 µm, respectively. The focal length was 8 mm with mechanical index (MI) = 0.2. Real-time imaging was performed at a frame rate of 10 Hz (corresponding to a temporal resolution of 100 ms). Two-dimensional B-mode image planes were acquired with optimization of the gain and the time-gain compensation settings, which were kept constant throughout the experiments. High-frequency US imaging was applied to the entire tumor of each mouse. After a radiologist identified the tumor lesions, about 1 × 10^8^ MBs (about 0.1 ml) were injected through the lateral tail vein and post-contrast-injection US imaging of the tumor lesions was immediately performed for 20 min.

**Fig. 2:**
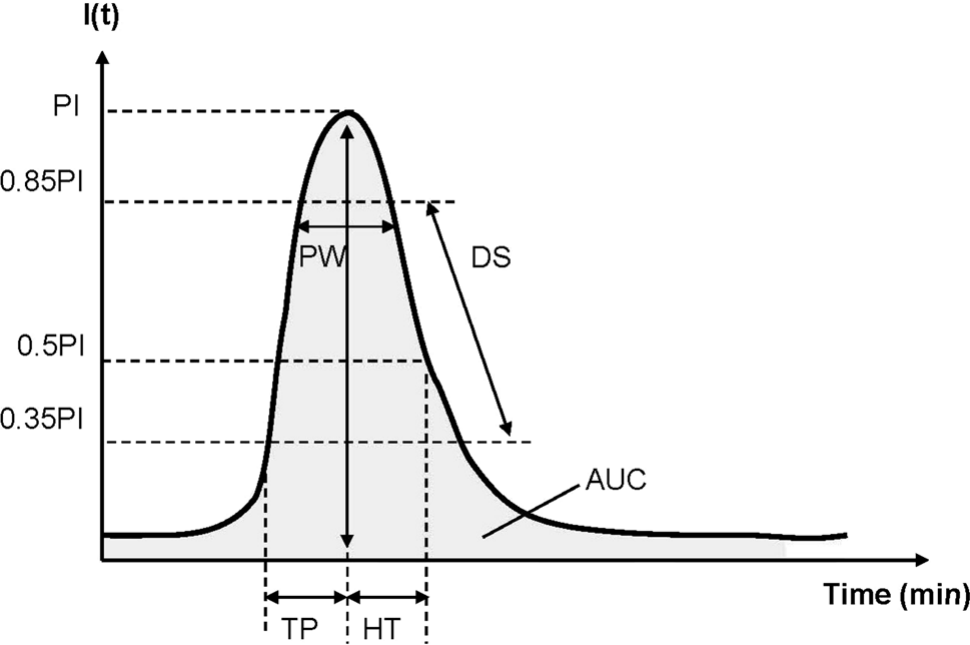
Schematic diagram of parameters for time intensity curve analysis (*top*). I(t) denotes intensity

The regions of interest (ROIs) were drawn over the whole tumor in a two-dimensional imaging plane by a radiologist, and the average pre- and post-contrast image intensities were measured in B-mode images. The images were stored digitally on a built-in hard drive for off-line analysis of the time-intensity relationship among the selected ROIs from the acquired image sections. The time-intensity curves (TICs) were then fitted using a least-squares algorithm based on the Nelder-Mead method (Matlab fminsearch function, Matlab, The MathWorks Inc., Natick, MA). Several parameters were derived from the TICs (Fig. 2): peak intensity (PI) was defined as the maximal intensity; time of peak intensity (TP) was the time required for the intensity to rise from the base to the peak corresponding to the MB injection time; half time (HT) was the time required for the intensity to decrease from the top to half of the maximum intensity; descending slope (DS) was the slope of a line between two specific points, i.e., 85 and 35 % of the peak intensity; peak width (PW) was the duration between of signal intensity maintaining higher than 85 % of peak intensity. Two points of 85 % of peak intensity, before and after the peak point; and area under the curve (AUC) was the summed area between the intensity curve and the time axis. Here AUC is in direct proportion to the amount of MBs present in the tumor.

### In Vivo Therapy by pUS and EGFR Antibody Delivery

Animals were anesthetized with isofluorane (2.5–3 % volume with oxygen). The animal was placed on the acrylic holder and the skin flanking the tumor was fastened and fixed by stainless clips. The top of the tumor was attached to the bottom of a filled water tank, which had a 50 × 50 mm^2^ opening. This opening was sealed with an acoustic-energy-permeable membrane and a 3-mm-thick gelatin pad to insure perfect coupling with no intervening gas. During sonication, mice were immobilized above the water tank with their tumor tightly attached to the thin-film window. The non-targeting US contrast agents were IV injected into the mice to facilitate acoustic cavitation. Since a wide sonication area is also helpful for increasing the permeability of tumor vessels, the focal zone of FUS was sequentially moved over the entire surface attached to the thin-film window, with the focus located 3 mm under the surface of the tumor.

A US transducer (focused type; diameter 60 mm; frequency 400 kHz) driven by the signal generated from an arbitrary function generator (33220A, Agilent, CA, USA) was used to enhance the permeability of tumor blood vessels. The FUS excitation signal was amplified by a radio-frequency power amplifier (150A100B, Amplifier Research) and monitored by a power-meter (Model 4421, Bird) before being fed into the US transducer.

pUS exposure (power 5 W; burst length 100 ms; pulse repetition frequency 1 Hz, sonication duration 20 s per exposure) in the presence of non-targeting MBs (mixed with 0.2 ml of saline, followed by flushing with 0.2 ml of heparin) was employed to enhance vascular permeability based on previous experiments on small animals [20–22]. Animals typically underwent 9–12 pUS exposures to allow coverage of the entire tumor region (range of total exposure time 180–240 s). Since the focal dimension was measured as approximately 3 mm along the radial direction, the spacing between individual adjacent focal positions was set at 3 mm.

**Fig. 3:**
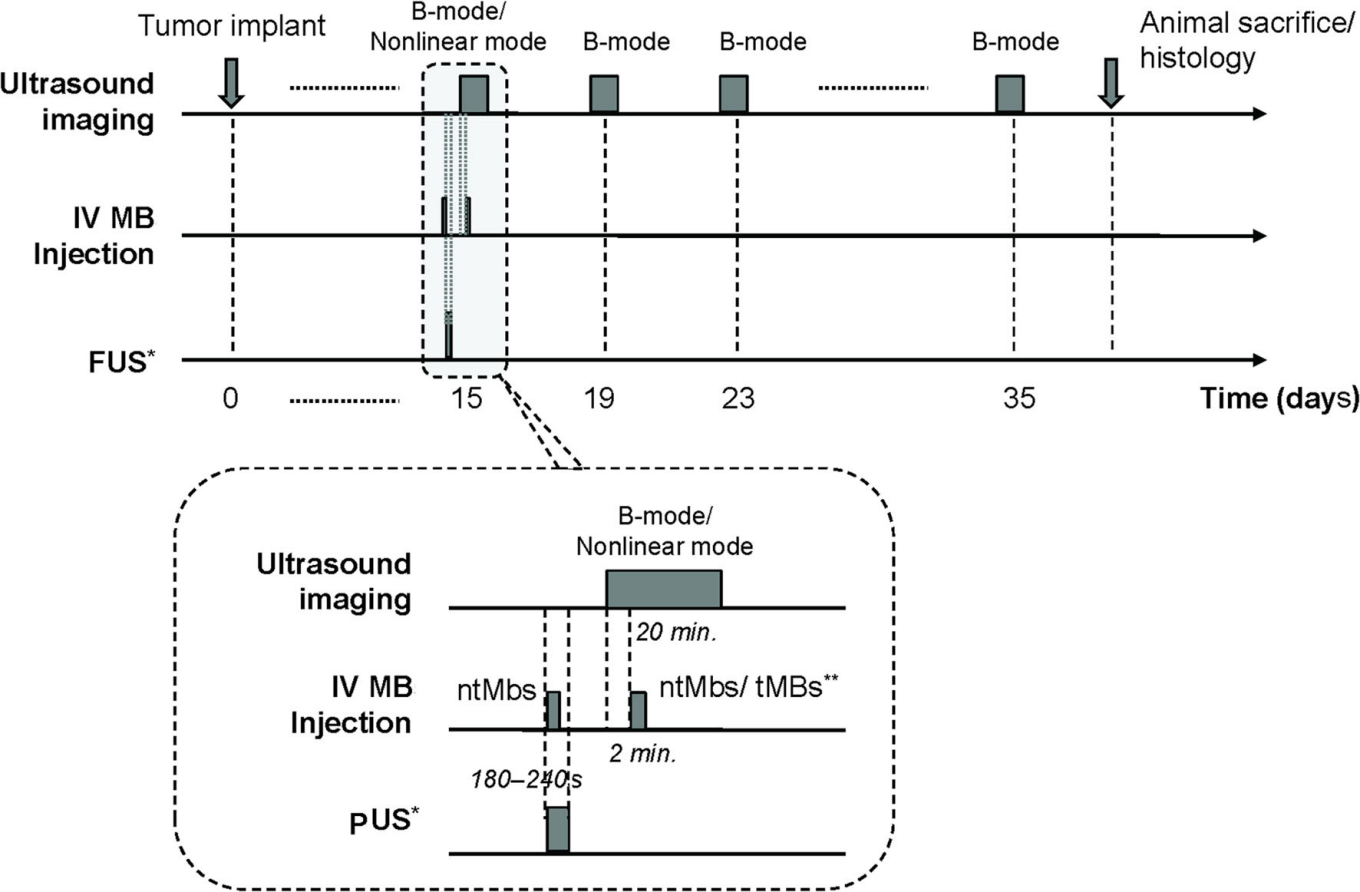
Experimental design and protocol (*bottom*). *only in group 3 animals; **non-targeting MBs were employed in group 1 animals, and targeting MBs were employed in group 2 and 3 animals

The tumor-bearing mice were divided randomly into three groups (*n* = 6 in each group): (1) IV-injected non-targeting Targestar™-SAMBs; (2) IV-injected targeting Targestar™-SA MBs; and (3) pUS exposure prior to IV injection of targeting Targestar™-SA MBs. Animals were treated on a daily basis with targeting Targestar™-SA MBs or pUS. During the experimental period of 35 days, tumor volumes were measured twice a week for growth curve measurements. At the end of the 35-day intervention, the mice were sacrificed and tumors were removed, rinsed in cold saline, and then patted between paper towels. A portion of each tumor was excised and fixed in 10 % paraformaldehyde overnight at room temperature. The tumor samples were embedded in paraffin for further immunohistochemical analysis. Detailed experimental designs are shown in Fig. 3.

### Estimation and Comparison of Tumor Growth

Vernier caliper measurements were made before and after treatment, in which the lengths of the three directions of the tumor and the tumor volume were calculated according to the ellipsoid model [21]. The doubling time is the period of time required for a quantity to double in size or value. Tumor doubling time was adopted as a factor to estimate tumor growth. The doubling time (*T*) of tumor cells in vivo was calculated as:

1 $$T = \frac{{(t - t_{0} ){\mkern 1mu} \times {\mkern 1mu} \log \;2}}{{\log \;N - \log \;N_{0} }}$$

where *t* is a time point during the exponential phase, *N* is the tumor volume at time *t*, and *N*_0_ is the tumor volume at initial time point *t*_0_ [23].

### Immunohistochemistry

Formalin-fixed, paraffin-embedded tissue samples from the surgically resected U87 tumor were cut into 4-mm sections. Sections were deparaffinized, blocked with 3 % H_2_O_2_ in methanol at 4 °C for 20 min, and rinsed with PBS. Sections were then blocked with normal goat serum for 30 min to increase the specificity of the primary antibody, and rinsed with PBS. Streptavidin peroxidase (Cat. No. TS-060-HR, Thermo Scientific) reagent was used as the primary antibody to estimate the delivery of injected biotin-modified therapeutic EGFR antibody. After blocking, the section was incubated overnight with 100-fold diluted primary antibody and rinsed with PBS. The directions of the Polymer-HRP IHC Detection System (QD420-YIK, BioGenex) were followed. Slides were then counterstained with hematoxylin and topped with a cover slip. Sections were also prepared for light microscopy by staining with hematoxylin and eosin (HE; Sigma-Aldrich, MO, USA) to assess overall tissue morphology and regional cell viability.

### Statistical Analysis

Data were analyzed statistically using Student’s *t* test. A probability value of *p* < 0.05 was considered statistically significant.

## Results

### In Vivo High-Frequency Ultrasound Glioma Tumor Imaging

**Fig. 4:**
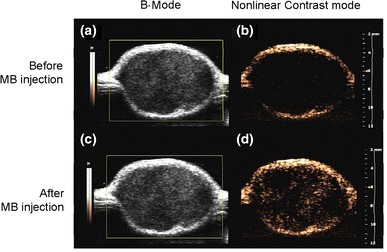
B-mode tumor images **a** before (pre-contrast) and **c** after IV injection of MBs. Nonlinear contrast mode tumor images **b** before and **d** after IV injection of MBs

**Fig. 5:**
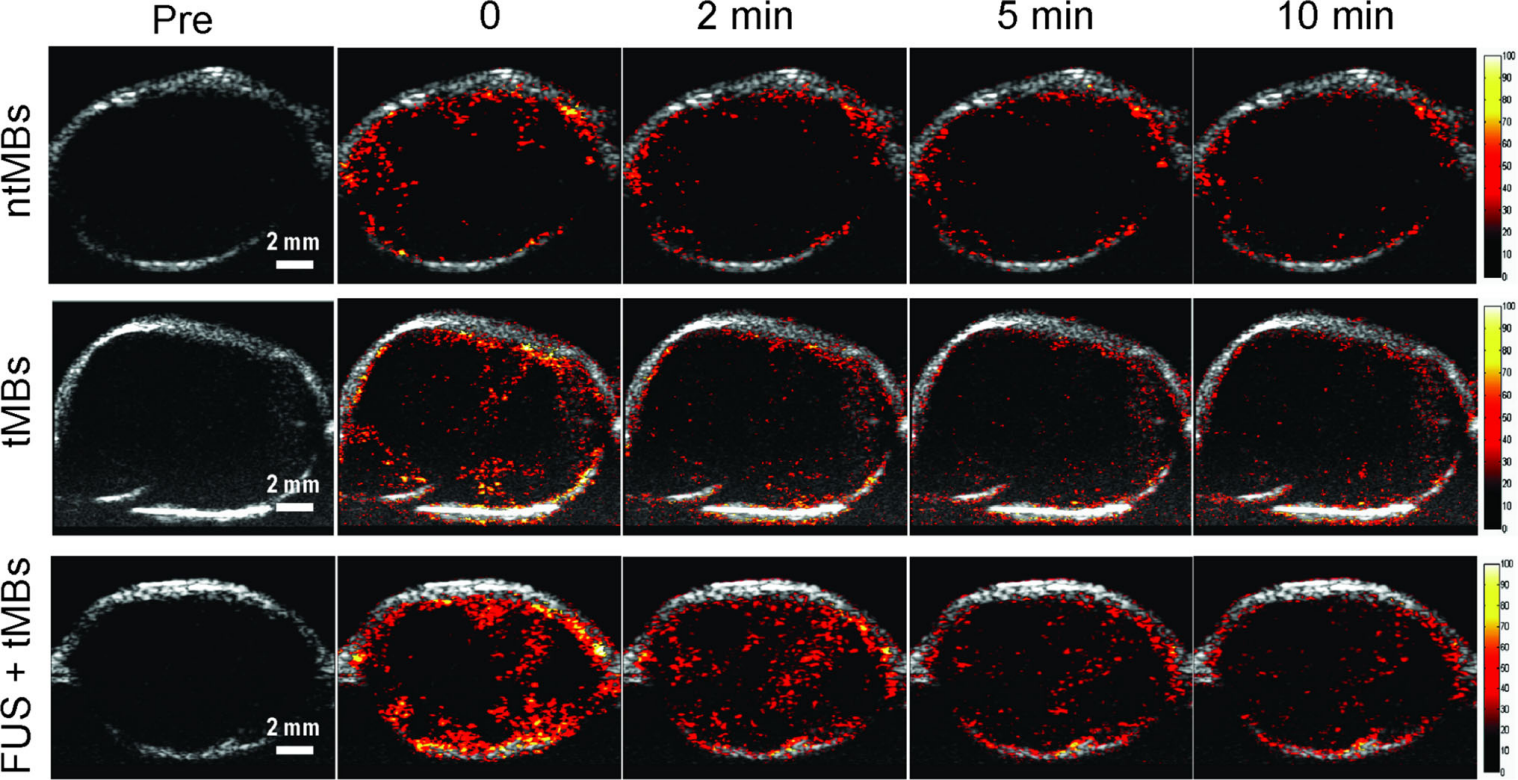
Contrast-enhanced US images of non-targeting MBs (*top row*), targeting MBs (*middle row*), and targeting MBs combined with pUS treatment (*bottom row*) at various times

**Fig. 6:**
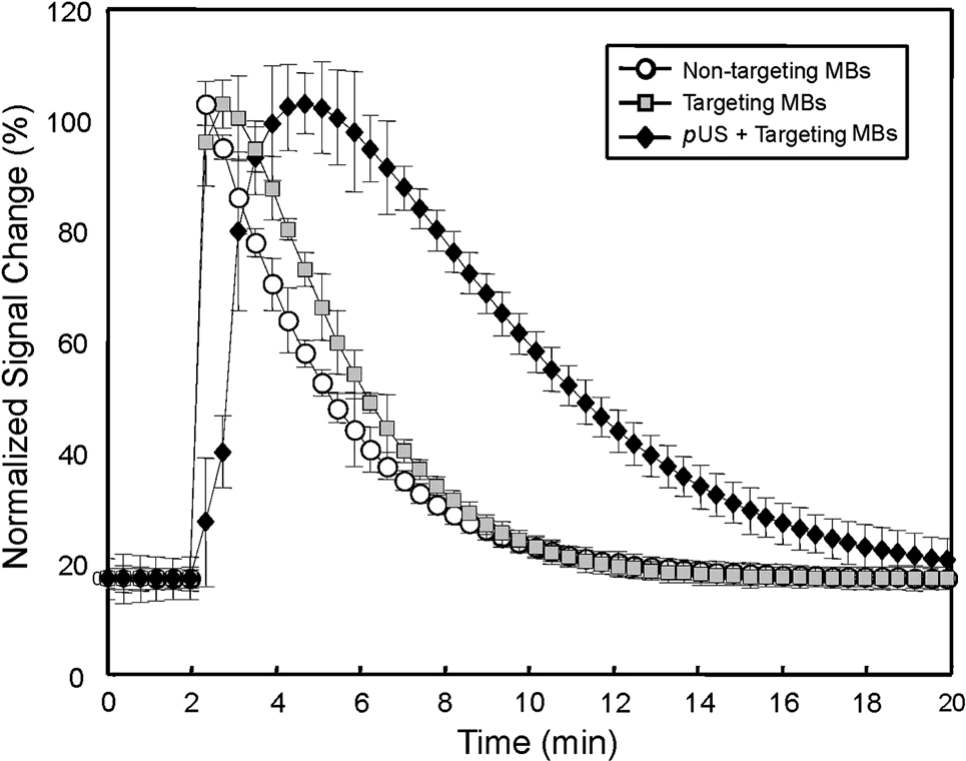
Typical TIC analysis of IV-injected non-targeting MBs, IV-injected targeting MBs, and IV-injected targeting MBs combined with pUS treatment

B-mode US images showed similar intensities before (pre-contrast) and after injection of MBs (Fig. 4a, c). However, the image intensity in nonlinear contrast mode was significantly higher after MB injection compared to that in pre-contrast images (Fig. 4b, d). Therefore, nonlinear contrast mode US imaging was used to investigate the accumulation of non-targeting MBs, targeting MBs, and targeting MBs combined with pUS treatment (Fig. 5). Image enhancement was lower in pre-contrast images (Fig. 5, column denoted as “pre”) compared to that in images of injected targeting or non-targeting MBs (Fig. 5, column denoted as “0”). Ten min after MB injection, image enhancement was still obvious for targeting MBs and targeting MBs with pUS treatment compared to that of non-targeting MBs. TICs were acquired for 20 min (Fig. 6) and used to calculate perfusion parameters (Table 1). The ratios of the perfusion parameters of the targeting and pUS-treated groups relative to that of the non-targeted MB group are shown in Table 2. The PI of non-targeting MBs was higher than those of the other two groups. However, the TP, HT, PW, and AUC values of targeting MBs with pUS treatment were higher than those of the other two groups. Thus, increased vascular permeability was observed for targeting MBs with pUS treatment. The DS of targeting MBs with pUS treatment was more moderate compared to those of the other two groups.

**Table 1:** Time intensity curve analysis using perfusion parameters

	Non-targeting MBs	Targeting MBs	Targeting MBs + pUS
PI	109.10	99.17	93.38
TP (min)	0.34	0.73	2.69
HT (min)	2.34	3.13	5.86
DS	−18.72	−17.42	−8.46
PW (min)	1.79	1.59	2.679
AUC	5,380.34	5,855.83	10,340.49

**Table 2:** Time intensity curve analysis using perfusion parameter ratios with respect to non-targeting MB group

	Targeting MBs	Targeting MBs + pUS
PI	0.91	0.86
TP (min)	2.13	7.81
HT (min)	1.33	2.50
DS	0.93	0.45
PW (min)	0.88	1.49
AUC	1.08	1.92

### In Vivo FUS Exposure with Therapeutic EGFR Antibody Delivery

**Fig. 7:**
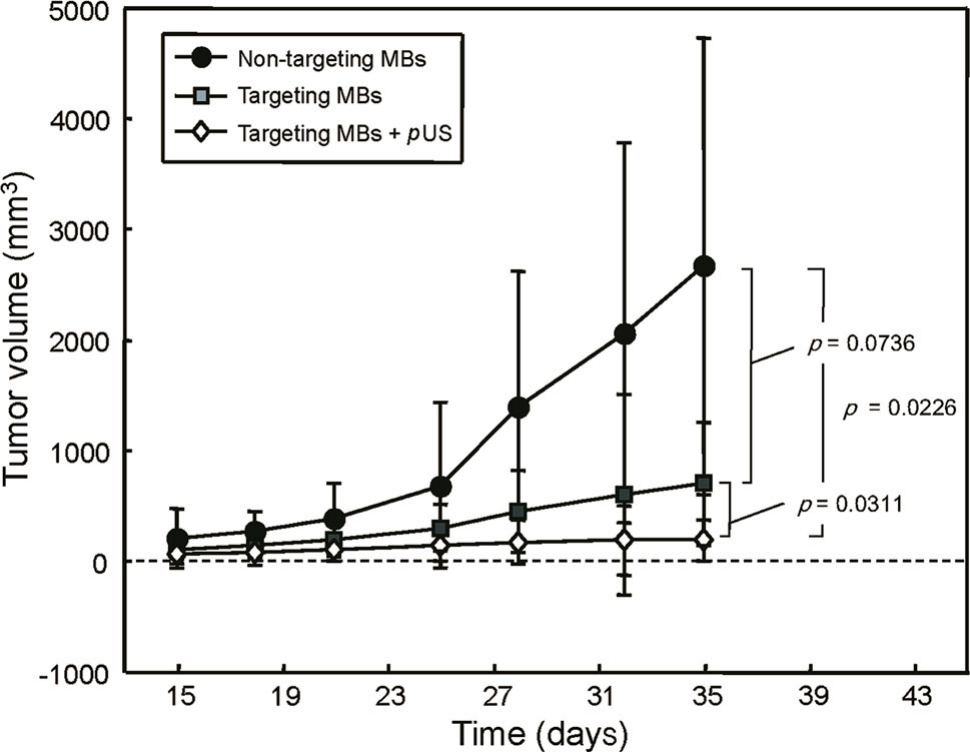
Tumor volume growth curves for treatment with non-targeting MBs, targeting MBs, and targeting MBs combined with pUS treatment. Targeting MBs after pUS treatment showed statistically significant growth suppression compared to all other groups (*p* < 0.05). Tumor growth of targeting MB treatment group was mildly suppressed compared to that of non-targeting MBs, but this difference was still statistically significant (*p* < 0.05)

**Fig. 8:**
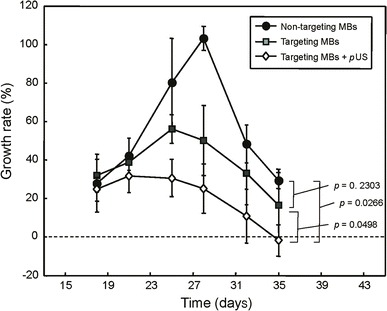
Tumor volume growth rates for treatment with non-targeting MBs, targeting MBs, and targeting MBs combined with pUS treatment

To evaluate the suppression of EGFR activity by EGFR antibody, tumor growth was observed for 35 days in the glioma-bearing mice. The growth curves and growth rates of U87 tumors for various treatments are shown in Figs. 7 and 8, respectively. Treatment with targeting MBs or targeting MBs after pUS treatment significantly reduced the growth of established U87 tumors. The final relative tumor volumes were 2,663.9 ± 2,058.3, 700.0 ± 557.9, and 188.4 ± 184.0 mm^3^ for non-targeting MBs, targeting MBs, and targeting MBs combined with pUS treatment, respectively (*p* value calculated by tumor volume: *P*_Targeting MBs_ = 0.0737 and *P*_Targeting MBs with pUS_ = 0.0226. *p* value calculated by tumor growth percentage: *P*_Targeting MBs_ = 0.2304 and *P*_Targeting MBs with pUS_ = 0.0267). These are equivalent to 38.2 and 209.8 % decreases in tumor doubling time and tumor volume, respectively, compared with those of control animals (Figs. 7 and 8). These results show that EGFR-targeting MBs injected after pUS treatment are sufficient for control of U87 tumors, presumably mediated by binding and inhibition of EGFR by EGFR antibody.

### Immunohistochemistry

**Fig. 9:**
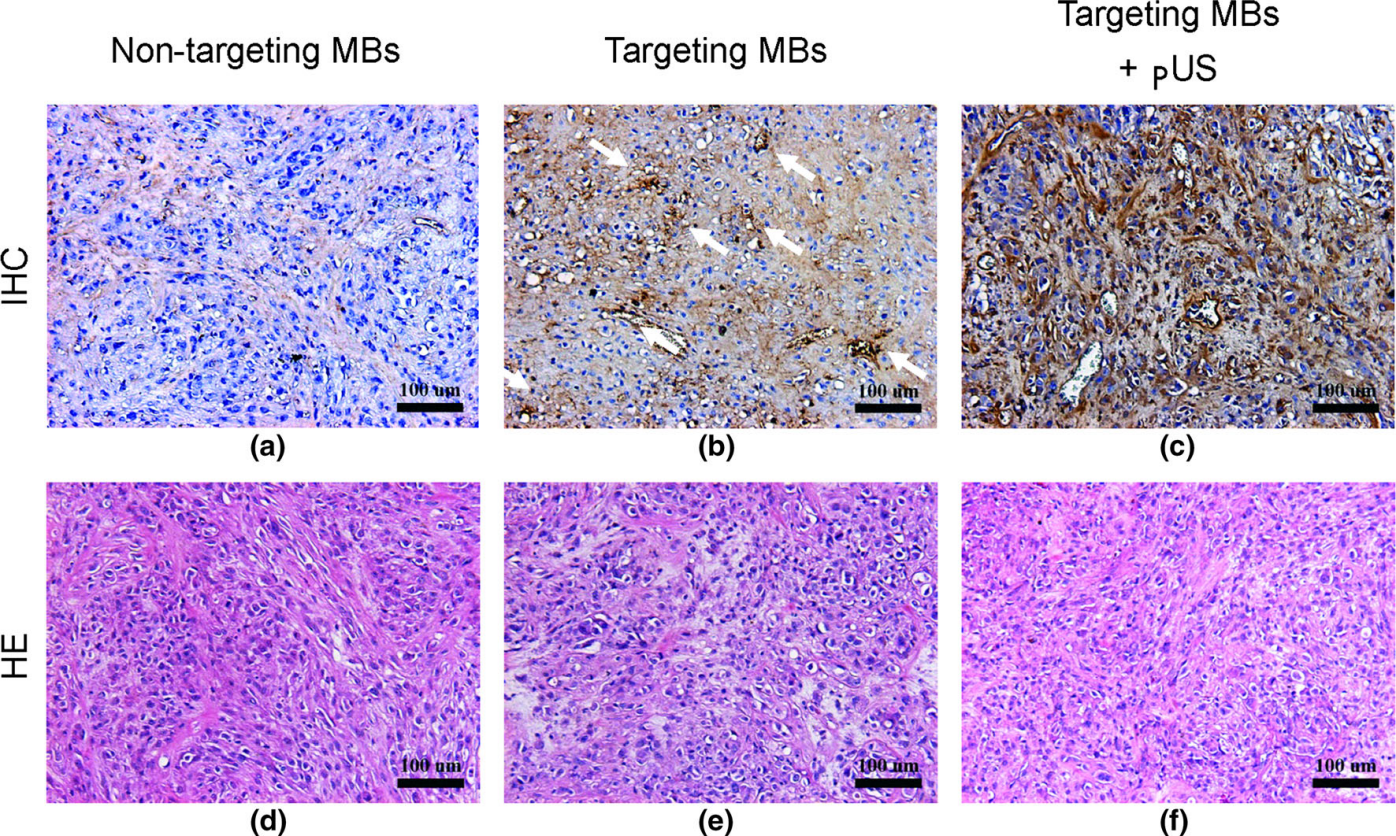
Histopathologic analysis. **a**–**c** Qualitative assessment of immunohistochemical staining (IHC; *upper row*) demonstrating low EGFR in non-targeting MB treatment group (**b**), and high EGFR intensity in tumor vessels of targeting MB treatment group in both tumor vessels and tissues of targeting MB combined with pUS treatment group (**c**); **d**–**f** HE staining revealing similar histology in all three treatment groups

EGFR antibody delivery by various treatments was evaluated by immunohistochemistry at 35 days (Fig. 9a–c). Sections were also stained with HE (Fig. 9d–f). EGFR antibody was not observed in the control group (Fig. 9a). Targeting MBs in the absence of pUS treatment resulted in high levels of EGFR antibody deposition, located mostly inside vessels. In the targeted MB with pUS treatment group, EGFR antibody leaked outside vessels and spread through tumor tissues (Fig. 9c). pUS treatment clearly enhanced drug delivery (Fig. 9b versus Fig. 9c), without major histological changes (HE stain; Fig. 9e versus Fig. 9f).

## Discussion

This study demonstrated the use of EGFR-targeting MBs combined with pUS exposure to enhance therapeutic EGFR antibody delivery to glioma tumor cells in mice. It was shown that pUS exposure increased the penetration and circulating half-life of targeting MBs. pUS exposure prior to injection of targeting MBs also led to a significantly better tumor suppressing effect. pUS exposure with MBs thus has potential for enhanced therapeutic antibody delivery for facilitating anti-glioma treatment.

Contrast-enhanced ultrasound (CEUS) has been widely used in the diagnosis of diseases of the heart, liver, kidney, pancreas, and peripheral vessels [24]. In this study, the potential of MB-facilitated pUS to enhance the delivery of therapeutic EGFR antibodies so that the process can be concurrently monitored by CEUS. Improved spatial resolution of nonlinear contrast imaging has been demonstrated previously [25]. The nonlinear contrast imaging mode on a micro-US system can be used to obtain parametric images to map various parameters related to blood flow in perfused tumors, with improved image contrast after the injection of MBs [26]. Such images were used here to generate TICs and determine values for various parameters (Tables 1 and 2). The TP, HT, and AUC values of non-targeting MBs were lower than those of both targeting MBs and targeting MBs with pUS. However, the PW of targeting MBs was lower than that of non-targeting MBs and targeting MBs with pUS. This result demonstrates the different perfusion characteristics of targeting MBs and targeting MBs with pUS. The image intensity for targeting MBs with pUS treatment exhibited a diffuse peak in the range of 3-6 min, remaining high until 10 min (Fig. 6). The shift of the TIC distribution in targeting MBs with pUS treatment indicate an increase in the lifetime of targeting MBs in the tumor and more targeting MBs delivered into the tumor.

EGFR therapy has focused mainly on blocking signal transduction with monoclonal antibodies. Many anti-EGFR monoclonal antibodies have been approved by the FDA for clinical treatment of head and neck cancer and colorectal cancer. However, all conventional small-molecule drugs or monoclonal antibodies are quickly metabolized and cleared through the kidneys, thus requiring high therapeutic concentrations, which in turn causes cardiotoxicity or other toxic side effects [24]. The present study shows that pUS treatment combined with therapeutic anti-EGFR antibodies enhances the anti-tumor effect in glioma-bearing mice. Although tumor growth was inhibited with targeting MB treatment in the absence of pUS, significant tumor growth suppression was observed with targeted MBs combined with pUS, mainly later after 7 days post treatment. Moreover, the tumors did not re-grow in the next 10 days, perhaps due to an enhanced anticancer effect by microvascular disruption caused by pUS exposure [22] and anti-EGFR monoclonal antibody treatment.

No immunological signal for EGFR antibody was observed in the control group in 35-day tumors. However, anti-EGFR monoclonal antibodies were detected within the tumor vessels in the targeting MB group, and in both tumor vessels and tissue in the targeting MB combined with pUS group. This treatment group showed both inhibition of EGFR-dependent glioma-cell growth and a large number of anti-EGFR monoclonal antibodies.

## Conclusion

In this study, the halftime of targeting MBs in glioma tumor was prolonged in glioma-bearing mice. Anti-EGFR therapy with targeting MBs inhibited the growth rate of glioma in BALB/c nude mice. pUS-mediated MB treatment increased tumor vessel permeability, thus increasing delivery of targeting MBs and significantly enhancing anti-EGFR therapy in the tumor. These results demonstrate the feasibility of tumor immunotherapy with targeting MBs combined with pUS.
